# Synthesis and Biological Evaluation of 2-Hydroxy-3-[(2-aryloxyethyl)amino]propyl 4-[(Alkoxycarbonyl)amino]benzoates

**DOI:** 10.1155/2013/274570

**Published:** 2013-10-29

**Authors:** Jan Tengler, Iva Kapustíková, Matúš Peško, Rodney Govender, Stanislava Keltošová, Petr Mokrý, Peter Kollár, Jim O'Mahony, Aidan Coffey, Katarína Král'ová, Josef Jampílek

**Affiliations:** ^1^Department of Chemical Drugs, Faculty of Pharmacy, University of Veterinary and Pharmaceutical Sciences, Palackého 1/3, 612 42 Brno, Czech Republic; ^2^Medis International a.s., Průmyslová 16, 747 23 Bolatice, Czech Republic; ^3^Department of Environmental Ecology, Faculty of Natural Sciences, Comenius University, Mlynska dolina Ch-2, 842 15 Bratislava, Slovakia; ^4^Department of Biological Sciences, Cork Institute of Technology, Bishopstown, Cork, Ireland; ^5^Department of Human Pharmacology and Toxicology, Faculty of Pharmacy, University of Veterinary and Pharmaceutical Sciences, Palackého 1/3, 612 42 Brno, Czech Republic; ^6^Institute of Chemistry, Faculty of Natural Sciences, Comenius University, Mlynská dolina CH-2, 842 15 Bratislava, Slovakia

## Abstract

A series of twenty substituted 2-hydroxy-3-[(2-aryloxyethyl)amino]propyl 4-[(alkoxycarbonyl)amino]benzoates were prepared and characterized. As similar compounds have been described as potential antimycobacterials, primary *in vitro* screening of the synthesized carbamates was also performed against two mycobacterial species. 2-Hydroxy-3-[2-(2,6-dimethoxyphenoxy)ethylamino]-propyl 4-(butoxycarbonylamino)benzoate hydrochloride, 2-hydroxy-3-[2-(4-methoxyphenoxy)ethylamino]-propyl 4-(butoxycarbonylamino)benzoate hydrochloride, and 2-hydroxy-3-[2-(2-methoxyphenoxy)ethylamino]-propyl 4-(butoxycarbonylamino)benzoate hydrochloride showed higher activity against *M. avium* subsp. *paratuberculosis* and *M. intracellulare* than the standards ciprofloxacin, isoniazid, or pyrazinamide. Cytotoxicity assay of effective compounds was performed using the human monocytic leukaemia THP-1 cell line. Compounds with predicted amphiphilic properties were also tested for their effects on the rate of photosynthetic electron transport (PET) in spinach (*Spinacia oleracea* L.) chloroplasts. All butyl derivatives significantly stimulated the rate of PET, indicating that the compounds can induce conformational changes in thylakoid membranes resulting in an increase of their permeability and so causing uncoupling of phosphorylation from electron transport.

## 1. Introduction

It is well known that the carbamate scaffold exhibits various biological effects and that the carbamate moiety (-NHCOO-) in the molecules is known to interact with a number of enzymes and biological structures [1, 2]. Carbamates are primarily known as local anaesthetics [3] and can also influence cardiovascular system functions [4, 5]. Nevertheless, *N*-benzoyl carbamate derivatives were also identified as potential antituberculotics [6–17].

Several authors have reported that tertiary amine local anesthetics decrease the value of the temperature of the gel-liquid crystalline phase transition *T*_*m*_ of model membranes, and this decrease correlated well with local anesthetic activity [18, 19]. A similar decrease of *T*_*m*_ was also observed in [2-(alkyloxy)-phenyl]-2-(l-piperidinyl)ethyl esters of carbamic acids [20]. The local anesthetic carbisocaine, a derivative of carbamic acid, was also found to exert a biphasic effect on the fluidity of egg yolk phosphatidylcholine (EYPC) model membranes as detected by the stearic acid spin probes with the paramagnetic doxyl group bound to C_(5)_ or C_(16)_. The fluidity initially increased with an increase in carbisocaine concentration, but at concentrations above 25 mmol/L a decrease of fluidity has been observed [21]. The results of a study using heptacaine, the monohydrochloride of [2-(heptyloxy)phenyl]-2-(1-piperidinyl)ethyl ester of carbamic acid, showed that the fluidity of EYPC model membranes initially increased as the molar ratio of heptacaine : EYPC increased, but at heptacaine : EYPC molar ratios above 0.5, a decrease of fluidity was observed [22]. This decrease in fluidity may be due to interdigitation of hydrocarbon chains in the bilayer. Based on the results of a study on influence of  2-piperidinoethyl-4-heptyloxyphenylcarbamate hydrochloride on metabolic function in *Staphylococcus aureus*, Mlynarčík et al. suggested that the bacteriostasis could be equated with a loss of the cell's ability to synthesize ATP, which, in turn, may stem from an uncoupling of oxidative phosphorylation [23]. Amphiphilic *N,N*-dimethylalkylamine *N*-oxides were found to modulate the activity of the purified sarcoplasmic reticulum (Ca-Mg)ATPase. The phase of insensitivity or slight stimulation of the activity at lower homologue concentrations was followed by the inhibition phase at higher concentrations [24].

As with other biological membranes under physiological conditions, the thylakoid membrane is almost impermeable to small charged molecules and ions. This property is essential for the maintenance of the electrochemical proton gradient generated by the photosynthetic electron transport chain, which serves as the driving force of ATP synthesis by ATP synthase. Consequently, alterations in membrane ion permeability are expected also to affect the efficiency of ATP production. Organic ammonium salts with local anaesthetic activity (e.g., dibucaine and tetracaine, which exhibit properties of protonophores) lower the proton gradient between the inside and the outside of the thylakoid membrane, causing loss of ability to form ATP [25]. Stimulation of oxygen evolution rate (OER) at low concentrations of surfactants was explained by the increase in the permeability of chloroplast envelope membrane or its destruction, resulting in the restraint of the phosphorylation system [26, 27] or by incorporation of a membrane active compound into the thylakoid membrane causing an increase of PET. *N*-Phenylcarbamates with R^1^ = 3,4-Cl_2_C_6_H_3_ and R^2^ = 4-NO_2_C_6_H_4_, CH_2_CHCl_2_, or CH_2_CF_3_ were found to be potent uncouplers which were able to fully uncouple the oxidative phosphorylation or the photophosphorylation between 1 and 10 *μ*mol/L. It was assumed that the -NH- group of the carbamate function is probably involved in the proton transfer through the thylakoid membranes [28]. Anthracene was also found to induce conformational changes in biomembranes resulting in an increase of their permeability, which was connected with ion leakage [29], and this modification of thylakoid membrane integrity led to uncoupling of phosphorylation from electron transport [30].

Based on previous interesting results of similar structures as new potential antituberculotics [12–17], a series of substituted *N*-arylcarbonyloxypropanol-*N*-aryloxyethyl-amines was synthesized, and selected physicochemical characteristics were described along with their antimycobacterial activity and cytotoxicity. The effects of the amphiphilic compounds on photosynthetic electron transport were also investigated.

## 2. Material and Methods

### 2.1. Chemistry

All reagents were purchased from Sigma-Aldrich in sufficient purity, and solvents were purchased from Lach-Ner and were dried if necessary. Kieselgel 60, 0.040–0.063 mm (Merck, Darmstadt, Germany) was used for column chromatography. TLC plates precoated by silica gel 60 F254 were used for reaction monitoring, and retardation factors *R*_*f*_ were determined by reversed-phase TLC glass plates DC Fertigplatten Merck RP-8 F254 S (both Merck, Darmstadt, Germany). The plates were illuminated under UV (254 nm). The melting points were measured on Kofler hot-plate apparatus HMK (Franz Kustner Nacht KG, Dresden, Germany) and are uncorrected. Infrared (IR) spectra were recorded on Nicolet iS5 FT-IR spectrometer (Thermo Scientific, USA) by ATR technique in the region of 4000–600 cm^−1^. The purity of the compounds was checked by HPLC separation module (Waters Alliance 2695 XE, Waters Corp., Milford, MA, USA). The detection wavelength 210 nm was chosen. Peaks in the chromatogram of the solvent (blank) were deducted from peaks in the chromatogram of the sample solution. Purity of the individual compounds was determined from peak area in the chromatogram of the sample solution. UV spectra (*λ*, nm) were determined on a Waters Photodiode Array Detector 2996 (Waters Corp., Milford, MA, USA) in ca 6 · 10^−4^ M methanolic solution. log⁡⁡*ε* (the logarithm of molar absorption coefficient *ε*) was calculated for the absolute maximum *λ*_max⁡_ of the individual compounds. ^1^H and ^13^C NMR spectra of products were recorded on a Bruker Avance 400 FT-NMR spectrometer (400 MHz for ^1^H and 100 MHz for ^13^C, Bruker Comp., Karlsruhe, Germany). ^1^H and ^13^C NMR spectra of some intermediates were recorded on a Gemini-2000 FT-NMR spectrometer (200 MHz for ^1^H and 50 MHz for ^13^C, Varian Inc., Palo Alto, USA). Chemical shifts are reported in ppm (*δ*). Proton and carbon chemical shifts in DMSO-*d*_6_ are related to the middle of the multiplet (*δ* = 2.50 and 39.5, resp.). ^13^C-NMR spectra were measured using APT pulse sequence. Coupling constants (*J*) are given in Hz. Mass spectra were measured using Agilent 1100 LC/MSD Trap (Agilent Technologies, USA) in positive mode.

#### 2.1.1. Synthesis

*Oxiran-2-ylmethyl-4-(alkoxycarbonylamino)benzoates *(**3a**–**d**). Oxiran derivatives **3a**–**d** were prepared by the method described by Mokrý et al. [5]. PCl_5_ was used to obtain chlorides (**2a**–**d**) from appropriate 4-(alkoxycarbonylamino) benzoic acids (**1a**–**d**) instead of thionyl chloride. Crude epoxides were recrystallized from isopropyl alcohol. Spectroscopic data were in agreement with the literature.

*2-Phenoxyethylamines *(**14**–**18**). Substituted aryloxyethyl bromides **4**–**8** were obtained according to a well-known alkylation as performed by Augstein et al. [31]. Resulting amines **14**–**18** were prepared via appropriate phthalimides **9**–**13** according to Reznik et al. [32]. 

*2-(2-Methoxyphenoxy)ethylamine *(**14**). Yield 58%; yellow oil; ^1^H NMR (200 MHz, DMSO-*d*_6_), *δ*: 6.98–6.84 (m, 4H, *Ar*), 3.88 (t, 2H, *J* = 5.9 Hz, OCH_2_), 3.75 (s, 3H, OCH_3_), 2.85 (t, 2H, *J* = 5.9 Hz, CH_2_N), 2.38 (bs, 2H, NH_2_); ^13^C NMR (50 MHz, DMSO-*d*_6_), *δ*: 149.2, 148.2, 120.9, 120.7, 113.7, 112.3, 79.1, 71.1, 55.4. 

*2-(4-Methoxyphenoxy)ethylamine *(**15**). Yield 75%; yellowish oil; ^1^H NMR (200 MHz, DMSO-*d*_6_), *δ*: 6.96–6.84 (m, 4H, *Ar*), 3.94 (t, 2H, *J* = 5.2 Hz, OCH_2_), 3.77 (s, 3H, OCH_3_), 3.05 (t, 2H, *J* = 5.2 Hz, NCH_2_), 1.58 (bs, 2H, NH_2_); ^13^C NMR (50 MHz, DMSO-*d*_6_), *δ*: 154.0, 153.2, 115.6, 114.8, 71.0, 55.7, 41.7. 

*2-(2,6-Dimethoxyphenoxy)ethylamine *(**16**). Yield 62%; yellow oil; ^1^H NMR (200 MHz, DMSO-*d*_6_), *δ*: 6.92 (t, 1H, *J* = 8.6 Hz, *p-Ar*), 6.54 (d, 2H, *J* = 8.6 Hz, *m-Ar*), 4.13 (t, 2H, *J* = 5.5 Hz, OCH_2_), 3.79 (s, 6H, OCH_3_), 3.65 (t, 2H, *J* = 5.5 Hz, CH_2_N), 1.95 (bs, 2H, NH_2_); ^13^C NMR (50 MHz, DMSO-*d*_6_), *δ*: 153.0, 138.1, 124.0, 105.8, 72.2, 55.9, 42.6. 

*2-(2-Fluorophenoxy)ethylamine *(**17**). Yield 82%; colourless oil; ^1^H NMR (200 MHz, DMSO-*d*_6_), *δ*: 7.29–7.10 (m, 2H, *Ar*), 7.04–6.93 (m, 2H, *Ar*), 4.28 (t, 2H, *J* = 5.2 Hz, CH_2_O), 3.14 (t, 2H, *J* = 5.2 Hz, CH_2_N), 1.91 (bs, 2H, NH_2_); ^13^C NMR (50 MHz, DMSO-*d*_6_), *δ*: 151.8 (d, *J*_CF_ = 244.2 Hz), 145.7 (d, *J*_CF_ = 10.5 Hz), 124.8 (d, *J*_CF_ = 3.8 Hz), 121.8 (d, *J*_CF_ = 6.9 Hz), 116.1 (d, *J*_CF_ = 17.9 Hz), 115.7, 65.6, 38.1. 

*2-(4-Fluorophenoxy)ethylamine *(**18**). Yield 79%; colourless oil; ^1^H NMR (200 MHz, DMSO-*d*_6_), *δ*: 7.04–7.17 (m, 2H, *Ar*F), 6.88–6.99 (m, 2H, *Ar*O), 3.87 (t, 2H, *J* = 5.8 Hz, OCH_2_), 2.85 (t, 2H, *J* = 5.8 Hz, CH_2_N), 1.97 (bs, 2H, NH_2_); ^13^C NMR (50 MHz, DMSO-*d*_6_), *δ*: 156.3 (d, *J*_CF_ = 235.5 Hz), 155.0 (d, *J*_CF_ = 1.8 Hz), 115.6 (d, *J* = 8.1 Hz), 115.6 (d, *J*_CF_ = 22.9 Hz), 70.8, 40.9. 

*4-(Alkoxycarbonylamino)-3-amino-2-hydroxypropyl Benzoate Derivatives *(**19a**–**d**, **20a**–**d**, **21a**–**d**, **22a**–**d**, **23a**–**d**). Intermediate **3a**–**d** (0.01 mol) was added to a solution of corresponding amine **14**–**18** (0.012 mol) in isopropyl alcohol (50 mL). Reaction was heated for 1 hour and stirred for 72 h at room temperature; then, the mixture was evaporated, and the crude basis product was dissolved in ethyl acetate and hydrochloride is transformed to its salt by addition of ethereal HCl. The obtained white precipitant was collected by filtration and recrystallized from isopropyl alcohol. The studied compounds are presented in Table 1. 

*2-Hydroxy-3-[2-(2-methoxyphenoxy)ethylamino]-propyl 4*-*(Methoxycarbonylamino)benzoate Hydrochloride *(**19a**). Yield 36%; m.p. 185*–*187°C; HPLC purity 94.74%; UV (nm), *λ*_max⁡_/log⁡⁡*ε* : 269.9/3.50; IR (Zn/Se ATR, cm^−1^): 3311, 2948, 2766, 1741, 1698, 1601, 1227; ^1^H NMR (400 MHz, DMSO-*d*_6_), *δ*: 10.12 (s, 1H, NHCO), 9.30 (bs, 1H, NH), 8.98 (bs, 1H, NH), 7.95 (d, 2H, *J* = 8.5 Hz, *Ar*COO), 7.61 (d, 2H, *J* = 8.5 Hz, *Ar*N), 7.05*–*6.88 (m, 4H, *Ar*OCH_3_), 5.99 (s, 1H, OH), 4.31*–*4.23 (m, 5H, CH_2_CHOH, CH_2_O), 3.75 (s, 3H, ArOCH_3_), 3.70 (s, 3H, COOCH_3_), 3.40*–*3.35 (m, 3H, CH_2_N, NCH_2_), 3.18*–*3.14 (m, 1H, CH_2_N); ^13^C NMR (100 MHz, DMSO-*d*_6_), *δ*: 165.1, 153.8, 149.4, 147.1, 143.9, 130.6, 122.9, 122.2, 120.7, 117.3, 114.9, 112.4, 65.9, 64.8, 64.4, 55.5, 51.9, 49.8, 46.1; MS: for C_21_H_27_N_2_O_7_ [M+H]^+^ calc. 419.174 m/z, found 419.3 m/z. 

*2-Hydroxy-3-[2-(2-methoxyphenoxy)ethylamino]-propyl 4*-*(Ethoxycarbonylamino)benzoate Hydrochloride *(**19b**). Yield 29%; m.p. 192*–*194°C; HPLC purity 96.76%; UV (nm), *λ*_max⁡_/log⁡⁡*ε* : 271.1/3.51; IR (Zn/Se ATR, cm^−1^): 3318, 2946, 2765, 1732, 1696, 1599, 1224; ^1^H NMR (400 MHz, DMSO-*d*_6_), *δ*: 10.08 (s, 1H, NHCO), 9.30 (bs, 1H, NH), 8.97 (bs, 1H, NH), 7.94 (d, 2H, *J* = 8.5 Hz, *Ar*COO), 7.61 (d, 2H, *J* = 8.5 Hz, *Ar*N), 7.05*–*6.88 (m, 4H, *Ar*OCH_3_), 5.96 (s, 1H, OH), 4.32–4.23 (m, 5H, CH_2_CHOH, CH_2_O), 4.16 (q, 2H, *J* = 7.1 Hz, COOCH_2_), 3.75 (s, 3H, ArOCH_3_), 3.40–3.34 (m, 3H, CH_2_N, NCH_2_), 3.18–3.14 (m, 1H, CH_2_N), 1.25 (t, 3H, *J* = 7.1 Hz, CH_3_); ^13^C NMR (100 MHz, DMSO-*d*_6_), *δ*: 165.1, 153.3, 149.4, 147.0, 144.0, 130.5, 122.8, 122.2, 120.7, 117.3, 114.9, 112.4, 65.9, 64.8, 64.4, 60.5, 55.5, 49.8, 46.1, 14.4; MS: for C_22_H_29_N_2_O_7_ [M+H]^+^ calc. 433.1897 m/z, found 433.3 m/z.

*2-Hydroxy-3-[2-(2-methoxyphenoxy)ethylamino]-propyl 4*-*(Propoxycarbonylamino)benzoate Hydrochloride* (**19c**). Yield 32%; m.p. 190–192°C; HPLC purity 98.01%; UV (nm), *λ*_max⁡_/log⁡⁡*ε* : 271.1/3.51; IR (Zn/Se ATR, cm^−1^): 3324, 2942, 2765, 1730, 1694, 1596, 1225; ^1^H NMR (400 MHz, DMSO-*d*_6_), *δ*: 10.08 (s, 1H, NHCO), 9.29 (bs, 1H, NH), 8.97 (bs, 1H, NH), 7.94 (d, 2H, *J* = 8.8 Hz, *Ar*COO), 7.61 (d, 2H, *J* = 8.8 Hz, *Ar*N), 7.05–6.88 (m, 4H, *Ar*OCH_3_), 5.96 (d, 1H, *J* = 4.5 Hz, OH), 4.32–4.23 (m, 5H, CH_2_CHOH, CH_2_O), 4.07 (t, 2H, *J* = 6.7 Hz, COOCH_2_), 3.75 (s, 3H, ArOCH_3_), 3.41–3.33 (m, 3H, CH_2_N, NCH_2_), 3.18–3.14 (m, 1H, CH_2_N), 1.69–1.61 (m, 2H, CH_3_CH_2_), 0.94 (t, 3H, *J* = 7.4 Hz, CH_3_); ^13^C NMR (100 MHz, DMSO-*d*_6_), *δ*: 165.1, 153.4, 149.4, 147.0, 144.0, 130.5, 122.8, 122.2, 120.7, 117.3, 114.9, 112.4, 66.0, 65.9, 64.8, 64.5, 55.5, 49.8, 46.1, 21.8, 10.2; MS: for C_23_H_31_N_2_O_7_ [M+H]^+^ calc. 447.2053 m/z, found 447.3 m/z. 

*2-Hydroxy-3-[2-(2-methoxyphenoxy)ethylamino]-propyl 4*-*(Butoxycarbonylamino)benzoate Hydrochloride* (**19d**). Yield 36%; m.p. 177–179°C; HPLC purity 98.79%; UV (nm), *λ*_max⁡_/log⁡⁡*ε* : 271.1/3.49; IR (Zn/Se ATR, cm^−1^): 3320, 2957, 2765, 1737, 1696, 1597, 1221; ^1^H NMR (400 MHz, DMSO-*d*_6_), *δ*: 10.07 (s, 1H, NHCO), 9.25 (bs, 1H, NH), 8.94 (bs, 1H, NH), 7.94 (d, 2H, *J* = 8.8 Hz, *Ar*COO), 7.61 (d, 2H, *J* = 8.8 Hz, *Ar*N), 7.05–6.88 (m, 4H, *Ar*OCH_3_), 5.95 (d, 1H, *J* = 3.5 Hz, OH), 4.32–4.23 (m, 5H, CH_2_CHOH, CH_2_O), 4.11 (t, 2H, *J* = 6.6 Hz, COOCH_2_), 3.75 (s, 3H, ArOCH_3_), 3.40–3.33 (m, 3H, CH_2_N, NCH_2_), 3.18–3.13 (m, 1H, CH_2_N), 1.64–1.59 (m, 2H, CH_2_CH_2_), 1.42–1.35 (m, 2H, CH_2_CH_2_), 0.92 (t, 3H, *J* = 7.4 Hz, CH_3_); ^13^C NMR (100 MHz, DMSO-*d*_6_), *δ*: 165.1, 153.4, 149.4, 147.0, 144.0, 130.5, 122.8, 122.2, 120.7, 117.3, 114.9, 112.4, 65.9, 64.8, 64.4, 64.2, 55.5, 49.8, 46.1, 30.5, 18.5, 13.5; MS: for C_24_H_33_N_2_O_7_ [M+H]^+^ calc. 461.2210 m/z, found 461.4 m/z.

*2-Hydroxy-3-[2-(4-methoxyphenoxy)ethylamino]-propyl 4*-*(Methoxycarbonylamino)benzoate Hydrochloride* (**20a**). Yield 43%; m.p. 194–195°C; HPLC purity 98.50%; UV (nm), *λ*_max⁡_/log⁡⁡*ε* : 271.1/3.48; IR (Zn/Se ATR, cm^−1^): 3322, 2952, 1740, 1704, 1601, 1229; ^1^H NMR (400 MHz, DMSO-*d*_6_), *δ*: 10.16 (s, 1H, NHCO), 9.31 (bs, 1H, NH), 9.04 (bs, 1H, NH), 7.95 (d, 2H, *J* = 8.5 Hz, *Ar*COO), 7.61 (d, 2H, *J* = 8.5 Hz, *Ar*N), 6.93–6.85 (m, 4H, *Ar*OCH_3_), 5.97 (s, 1H, OH), 4.31–4.20 (m, 5H, CH_2_CHOH, CH_2_O), 3.70 (s, 3H, ArOCH_3_), 3.69 (s, 3H, COOCH_3_), 3.31–3.26 (m, 3H, CH_2_N, NCH_2_), 3.14–3.08 (m, 1H, CH_2_N); ^13^C NMR (100 MHz, DMSO-*d*_6_), *δ*: 165.1, 153.8, 153.4, 151.7, 143.7, 130.6, 122.9, 117.3, 115.9, 114.6, 65.9, 64.5, 63.9, 55.4, 52.0, 49.8, 46.2; MS: for C_21_H_27_N_2_O_7_ [M+H]^+^ calc. 419.174 m/z, found 419.3 m/z.

*2-Hydroxy-3-[2-(4-methoxyphenoxy)ethylamino]-propyl 4-(Ethoxycarbonylamino)benzoate Hydrochloride* (**20b**). Yield 60%; m.p. 198–200°C; HPLC purity 98.21%; UV (nm), *λ*_max⁡_/log⁡⁡*ε* : 271.1/3.49; IR (Zn/Se ATR, cm^−1^): 3331, 2979, 1735, 1703, 1599, 1226; ^1^H NMR (400 MHz, DMSO-*d*_6_), *δ*: 10.11 (s, 1H, NHCO), 9.26 (bs, 1H, NH), 9.01 (bs, 1H, NH), 7.94 (d, 2H, *J* = 8.8 Hz, *Ar*COO), 7.61 (d, 2H, *J* = 8.8 Hz, *Ar*N), 6.93–6.86 (m, 4H, *Ar*OCH_3_), 5.96 (s, 1H, OH), 4.27–4.22 (m, 5H, CH_2_CHOH, CH_2_O), 4.15 (q, 2H, *J* = 7.1 Hz, COOCH_2_), 3.70 (s, 3H, ArOCH_3_), 3.32–3.24 (m, 3H, CH_2_N, NCH_2_), 3.16–3.07 (m, 1H, CH_2_N), 1.25 (t, 3H, *J* = 7.1 Hz, CH_3_); ^13^C NMR (100 MHz, DMSO-*d*_6_), *δ*: 165.2, 153.8, 153.3, 151.7, 144.0, 130.6, 122.9, 117.3, 115.7, 114.6, 65.9, 64.5, 63.9, 60.6, 55.4, 49.8, 46.2, 14.4, MS: for C_22_H_29_N_2_O_7_ [M+H]^+^ calc. 433.1897 m/z, found 433.3 m/z.

*2-Hydroxy-3-[2-(4-methoxyphenoxy)ethylamino]-propyl 4-(Propoxycarbonylamino)benzoate Hydrochloride* (**20c**). Yield 56%; m.p. 192–194°C; HPLC purity 98.31%; UV (nm), *λ*_max⁡_/log⁡⁡*ε* : 271.1/3.47; IR (Zn/Se ATR, cm^−1^): 3319, 2969, 2765, 1735, 1696, 1607, 1224; ^1^H NMR (400 MHz, DMSO-*d*_6_), *δ*: 10.17 (s, 1H, NHCO), 9.33 (s, 1H, NH), 9.06 (s, 1H, NH), 7.94 (d, 2H, *J* = 8.8 Hz, *Ar*COO), 7.62 (d, 2H, *J *= 8.8 Hz, *Ar*N), 6.93–6.85 (m, 4H, *Ar*OCH_3_), 5.97 (s, 1H, OH), 4.27–4.22 (m, 5H, CH_2_CHOH, CH_2_O), 4.06 (t, 2H, *J* = 6.7 Hz, COOCH_2_), 3.70 (s, 3H, ArOCH_3_), 3.33–3.27 (m, 3H, CH_2_N, NCH_2_), 3.16–3.08 (m, 1H, CH_2_N), 1.69–1.60 (m, 2H, CH_3_CH_2_), 0.93 (t, 3H, *J* = 7.4 Hz, CH_3_); ^13^C NMR (100 MHz, DMSO-*d*_6_), *δ*: 165.2, 153.8, 153.5, 151.7, 144.1, 130.6, 122.9, 117.3, 115.7, 114.6, 66.1, 65.9, 64.5, 63.9, 55.4, 49.8, 46.2, 21.8, 10.3; MS: for C_23_H_31_N_2_O_7_ [M+H]^+^ calc. 447.2053 m/z, found 447.3 m/z. 

*2-Hydroxy-3-[2-(4-methoxyphenoxy)ethylamino]-propyl 4-(Butoxycarbonylamino)benzoate Hydrochloride* (**20d**). Yield 35%; m.p. 190–192°C; HPLC purity 98.94%; UV (nm), *λ*_max⁡_/log⁡⁡*ε* : 271.1/3.46; IR (Zn/Se ATR, cm^−1^): 3327, 2964, 1734, 1700, 1597, 1223; ^1^H NMR (400 MHz, DMSO-*d*_6_), *δ*: 10.11 (s, 1H, NHCO), 9.34 (bs, 1H, NH), 9.07 (bs, 1H, NH), 7.94 (d, 2H, *J* = 8.6 Hz, *Ar*COO), 7.62 (d, 2H, *J* = 8.6 Hz, *Ar*N), 6.93–6.81 (m, 4H, *Ar*OCH_3_), 5.97 (d, 1H, *J* = 3.5 Hz, OH), 4.31–4.20 (m, 5H, CH_2_CHOH, CH_2_O), 4.11 (t, 2H, *J* = 6.5 Hz, COOCH_2_), 3.70 (s, 3H, ArOCH_3_), 3.33–3.25 (m, 3H, CH_2_N, NCH_2_), 3.15–3.07 (m, 1H, CH_2_N), 1.65–1.58 (m, 2H, CH_2_CH_2_), 1.43–1.33 (m, 2H, CH_2_CH_2_), 0.91 (t, 3H, *J* = 7.4 Hz, CH_3_); ^13^C NMR (100 MHz, DMSO-*d*_6_), *δ*: 165.2, 153.8, 153.4, 151.7, 144.0, 130.6, 122.9, 117.3, 115.7, 114.6, 65.9, 64.5, 64.2, 63.9, 55.3, 49.8, 46.2, 30.5, 18.6, 13.6; MS: for C_24_H_33_N_2_O_7_ [M+H]^+^ calc. 461.2210 m/z, found 461.4 m/z. 

*2-Hydroxy-3-[2-(2,6-dimethoxyphenoxy)ethylamino]-propyl 4-(Methoxycarbonylamino)benzoate Hydrochloride* (**21a**). Yield 42%; m.p. 202–204°C; HPLC purity 98.86%; UV (nm), *λ*_max⁡_/log⁡⁡*ε* : 268.7/3.49; IR (Zn/Se ATR, cm^−1^): 3302, 2951, 2622, 1745, 1695, 1599, 1227, 1102; ^1^H NMR (400 MHz, DMSO-*d*_6_), *δ*: 10.15 (s, 1H, NHCO), 9.31 (bs, 1H, NH), 8.59 (bs, 1H, NH), 7.95 (d, 2H, *J* = 8.8 Hz, *Ar*COO), 7.60 (d, 2H, *J* = 8.8 Hz, *Ar*N), 7.06 (t, 1H, *J* = 8.6 Hz, *p-Ar*OCH_3_), 6.70 (d, 2H, *J* = 8.6 Hz, *m-Ar*OCH_3_), 6.06 (s, 1H, OH), 4.32–4.22 (m, 3H, CH_2_CHOH), 4.17–4.14 (m, 2H, CH_2_O), 3.79 (s, 6H, ArOCH_3_), 3.69 (s, 3H, COOCH_3_), 3.40–3.33 (m, 3H, CH_2_N, NCH_2_), 3.29–3.26 (m, 1H, CH_2_N); ^13^C NMR (100 MHz, DMSO-*d*_6_), *δ*: 165.2, 153.8, 153.0, 145.0, 135.2, 130.6, 124.5, 122.9, 117.3, 105.3, 67.9, 66.0, 64.5, 55.9, 52.0, 49.3, 46.8; MS: for C_22_H_29_N_2_O_8_ [M+H]^+^ calc. 449.1846 m/z, found 449.3 m/z. 

*2-Hydroxy-3-[2-(2,6-dimethoxyphenoxy)ethylamino]-propyl 4-(Ethoxycarbonylamino)benzoate Hydrochloride* (**21b**). Yield 20%; m.p. 215–217°C; HPLC purity 98.07%; UV (nm), *λ*_max⁡_/log⁡⁡*ε* : 269.9/3.48; IR (Zn/Se ATR, cm^−1^): 3311, 2945, 2620, 1741, 1694, 1596, 1216, 1100; ^1^H NMR (400 MHz, DMSO-*d*_6_), *δ*: 10.11 (s, 1H, NHCO), 9.39 (bs, 1H, NH), 8.62 (bs, 1H, NH), 7.95 (d, 2H, *J* = 8.7 Hz, *Ar*COO), 7.60 (d, 2H, *J* = 8.7 Hz, *Ar*N), 7.05 (t, 1H, *J* = 8.4 Hz, *p-Ar*OCH_3_), 6.70 (d, 2H, *J* = 8.4 Hz, *m-*ArOCH_3_), 6.08 (d, 1H, *J* = 4.5 Hz, OH), 4.32–4.22 (m, 3H, CH_2_CHOH), 4.17–4.12 (m, 4H, COOCH_2_, CH_2_O), 3.79 (s, 6H, ArOCH_3_), 3.36–3.27 (m, 3H, CH_2_N, NCH_2_), 3.21–3.16 (m, 1H, CH_2_N), 1.25 (t, 3H, *J* = 7.1 Hz, CH_3_); ^13^C NMR (100 MHz, DMSO-*d*_6_), *δ*: 165.2, 153.3, 153.0, 145.0, 135.2, 130.6, 124.5, 122.9, 117.3, 105.3, 67.9, 66.0, 64.5, 60.6, 55.9, 49.3, 46.7, 14.4; MS: for C_23_H_31_N_2_O_8_ [M+H]^+^ calc. 463.2002 m/z, found 463.3 m/z. 

*2-Hydroxy-3-[2-(2,6-dimethoxyphenoxy)ethylamino]-propyl 4-(Propoxycarbonylamino)benzoate Hydrochloride* (**21c**). Yield 36%; m.p. 195–197°C; HPLC purity 98.37%; UV (nm), *λ*_max⁡_/log⁡⁡*ε* : 269.9/3.50; IR (Zn/Se ATR, cm^−1^): 3352, 2969, 2619, 1736, 1697, 1597, 1219, 1105; ^1^H NMR (400 MHz, DMSO-*d*_6_), *δ*: 10.10 (s, 1H, NHCO), 9.31 (bs, 1H, NH), 8.60 (bs, 1H, NH), 7.95 (d, 2H, *J* = 8.8 Hz, *Ar*COO), 7.60 (d, 2H, *J* = 8.8 Hz, *Ar*N), 7.06 (t, 1H, *J* = 8.4 Hz, *p-Ar*OCH_3_), 6.70 (d, 2H, *J* = 8.4 Hz, *m-*ArOCH_3_), 6.05 (s, 1H, OH), 4.33–4.21 (m, 3H, CH_2_CHOH), 4.18–4.13 (m, 2H, CH_2_O), 4.06 (t, 2H, *J* = 6.7 Hz, COOCH_2_), 3.79 (s, 6H, ArOCH_3_), 3.42–3.21 (m, 4H, CH_2_N, NCH_2_), 1.74–1.56 (m, 2H, CH_3_CH_2_), 0.93 (t, 3H, *J* = 7.4 Hz, CH_3_); ^13^C NMR (100 MHz, DMSO-*d*_6_), *δ*: 165.2, 153.4, 153.0, 144.0, 135.2, 130.6, 124.5, 122.9, 117.3, 105.3, 67.9, 66.0, 65.9, 64.5, 55.9, 49.3, 46.7, 21.8, 10.2; MS: for C_24_H_33_N_2_O_8_ [M+H]^+^ calc. 477.2159 m/z, found 477.4 m/z. 

*2-Hydroxy-3-[2-(2,6-dimethoxyphenoxy)ethylamino]-propyl 4-(Butoxycarbonylamino)benzoate Hydrochloride* (**21d**). Yield 34%; m.p. 188–190°C; HPLC purity 98.74%; UV (nm), *λ*_max⁡_/log⁡⁡*ε* : 269.9/3.48; IR (Zn/Se ATR, cm^−1^): 3305, 2960, 2623, 1739, 1684, 1598, 1219, 1103; ^1^H NMR (400 MHz, DMSO-*d*_6_), *δ*: 10.09 (s, 1H, NHCO), 9.41 (bs, 1H, NH), 8.63 (bs, 1H, NH), 7.95 (d, 2H, *J* = 8.8 Hz, *Ar*COO), 7.61 (d, 2H, *J* = 8.8 Hz, *Ar*N), 7.05 (t, 1H, *J* = 8.4 Hz, *p-Ar*OCH_3_), 6.70 (d, 2H, *J* = 8.4 Hz, *m-*ArOCH_3_), 6.07 (d, 1H, *J* = 4.5 Hz, OH), 4.33–4.23 (m, 3H, CH_2_CHOH), 4.18–4.15 (m, 2H, CH_2_O), 4.10 (t, 2H, *J* = 6.6 Hz, COOCH_2_), 3.79 (s, 6H, ArOCH_3_), 3.36–3.28 (m, 3H, CH_2_N, NCH_2_), 3.21–3.16 (m, 1H, CH_2_N), 1.64–1.57 (m, 2H, CH_2_CH_2_), 1.42–1.33 (m, 2H, CH_3_CH_2_), 0.91 (t, 3H, *J* = 7.4 Hz, CH_3_); ^13^C NMR (100 MHz, DMSO-*d*_6_), *δ*: 165.2, 153.4, 153.0, 144.0, 135.2, 130.6, 124.5, 122.9, 117.3, 105.3, 67.9, 65.9, 64.5, 64.2, 55.9, 49.3, 46.7, 30.5, 18.6, 13.6; MS: for C_25_H_35_N_2_O_8_ [M+H]^+^ calc. 491.2315 m/z, found 491.4 m/z. 

*3-[2-(2-Fluorophenoxy)ethylamino]-2-hydroxypropyl 4-(Methoxycarbonylamino)benzoate Hydrochloride* (**22a**). Yield 76%; m.p. 195–197°C; HPLC purity 98.91%; UV (nm), *λ*_max⁡_/log⁡⁡*ε* : 268.7/3.48; IR (Zn/Se ATR, cm^−1^): 3321, 2953, 2766, 1744, 1698, 1601, 1229; ^1^H NMR (400 MHz, DMSO-*d*_6_), *δ*: 10.15 (s, 1H, NHCO), 9.44 (bs, 1H, NH), 9.11 (bs, 1H, NH), 7.94 (d, 2H, *J* = 8.8 Hz, *Ar*COO), 7.61 (d, 2H, *J* = 8.8 Hz, *Ar*N), 7.26–7.14 (m, 2H, *Ar*F), 7.02–6.97 (m, 2H, *Ar*F), 5.98 (s, 1H, OH), 4.43–4.40 (m, 2H, CH_2_O), 4.28–4.23 (m, 3H, CH_2_CHOH), 3.69 (s, 3H, CH_3_), 3.56–3.43 (m, 2H, NCH_2_), 3.38–3.30 (m, 1H, CH_2_N), 3.17–3.11 (m, 1H, CH_2_N); ^13^C NMR (100 MHz, DMSO-*d*_6_), *δ*: 165.1, 153.8, 151.7 (d, *J*_CF_ = 243.9 Hz), 145.60 (d, *J*_CF_ = 10.4 Hz), 143.9, 130.6, 124.9 (d, *J*_CF_ = 3.8 Hz), 122.9, 121.9 (d, *J*_CF_ = 6.6 Hz), 117.3, 116.2 (d, *J*_CF_ = 17.7 Hz), 115.4, 65.9, 64.6, 64.5, 51.9, 49.9, 46.0; MS: for C_20_H_24_FN_2_O_6_ [M+H]^+^ calc. 407.1540 m/z, found 407.3 m/z. 

*3-[2-(2-Fluorophenoxy)ethylamino]-2-hydroxypropyl 4-(Ethoxycarbonylamino)benzoate Hydrochloride* (**22b**). Yield 54%; m.p. 209–211°C; HPLC purity 98.14%; UV (nm), *λ*_max⁡_/log⁡⁡*ε* : 269.9/3.48; IR (Zn/Se ATR, cm^−1^): 3327, 2961, 2759, 1733, 1694, 1599, 1221; ^1^H NMR (400 MHz, DMSO-*d*_6_), *δ*: 10.11 (s, 1H, NHCO), 9.44 (bs, 1H, NH), 9.11 (bs, 1H, NH), 7.94 (d, 2H, *J* = 8.4 Hz, *Ar*COO), 7.61 (d, 2H, *J* = 8.4 Hz, *Ar*N), 7.26–7.14 (m, 2H, *Ar*F), 7.02–6.97 (m, 2H, *Ar*F), 5.98 (d, 1H, *J* = 3.4 Hz, OH), 4.46–4.38 (m, 2H, CH_2_O), 4.27–4.23 (m, 3H, CH_2_CHOH), 4.15 (q, 2H, *J* = 7.1 Hz, COOCH_2_), 3.48–3.41 (m, 2H, NCH_2_), 3.37–3.30 (m, 1H, CH_2_N), 3.17–3.11 (m, 1H, CH_2_N), 1, 25 (t, 3H, *J* = 7.1, CH_3_); ^13^C NMR (100 MHz, DMSO-*d*_6_), *δ*: 165.2, 153.3, 151.7 (d, *J*_CF_ = 244.0 Hz), 145.6 (d, *J*_CF_ = 10.5 Hz), 144.0, 130.6, 124.9 (d, *J*_CF_ = 3.7 Hz), 122.9, 121.9 (d, *J* = 6.8 Hz), 117.3, 116.2 (d, *J*_CF_ = 17.8 Hz), 115.4, 65.9, 64.6, 64.5, 60.6, 49.9, 46.0, 14.4; MS: for C_21_H_26_FN_2_O_6_ [M+H]^+^ calc. 421.1697 m/z, found 421.3 m/z. 

*3-[2-(2-Fluorophenoxy)ethylamino]-2-hydroxypropyl 4-(Propoxycarbonylamino)benzoate Hydrochloride* (**22c**). Yield 53%; m.p. 201–203°C; HPLC purity 98.48%; UV (nm), *λ*_max⁡_/log⁡⁡*ε* : 268.7/3.50; IR (Zn/Se ATR, cm^−1^): 3328, 2956, 2754, 1731, 1695, 1599, 1221; ^1^H NMR (400 MHz, DMSO-*d*_6_), *δ*: 10.11 (s, 1H, NHCO), 9.43 (bs, 1H, NH), 9.10 (bs, 1H, NH), 7.94 (d, 2H, *J* = 8.7 Hz, *Ar*COO), 7.61 (d, 2H, *J* = 87 Hz, *Ar*N), 7.26–7.14 (m, 2H, *Ar*F), 7.02–6.97 (m, 2H, *Ar*F), 5.97 (s, 1H, OH), 4.43–4.40 (m, 2H, CH_2_O), 4.27–4.23 (m, 3H, CH_2_CHOH), 4.06 (t, 2H, *J* = 6.6 Hz, COOCH_2_), 3.46–3.43 (m, 2H, NCH_2_), 3.36–3.30 (m, 1H, CH_2_N), 3.17–3.11 (m, 1H, CH_2_N), 1.65–1.57 (m, 2H, CH_3_CH_2_), 0.93 (t, 3H, *J* = 7.4 Hz, CH_3_); ^13^C NMR (100 MHz, DMSO-*d*_6_), *δ*: 165.1, 153.4, 151.7 (d, *J*_CF_ = 244.0 Hz), 145.6 (d, *J*_CF_ = 10.5 Hz), 144.0, 130.6, 124.9 (d, *J* = 4.0 Hz), 122.9, 121.9 (d, *J*_CF_ = 7.0 Hz), 117.3, 116.2 (d, *J*_CF_ = 17.8 Hz), 115.4, 66.1, 65.9, 64.6, 64.5, 49.9, 46.0, 21.8, 10,3; MS: for C_22_H_28_FN_2_O_6_ [M+H]^+^ calc. 435.1853 m/z, found 435.3 m/z. 

*3-[2-(2-Fluorophenoxy)ethylamino]-2-hydroxypropyl 4-(Butoxycarbonylamino)benzoate Hydrochloride* (**22d**). Yield 36%; m.p. 191–192°C; HPLC purity 98.94%; UV (nm), *λ*_max⁡_/log⁡⁡*ε* : 269.9/3.49; IR (Zn/Se ATR, cm^−1^): 3330, 2971, 2750, 1732, 1695, 1597, 1226; ^1^H NMR (400 MHz, DMSO-*d*_6_), *δ*: 10.10 (s, 1H, NHCO), 9.45 (bs, 1H, NH), 9.13 (bs, 1H, NH), 7.94 (d, 2H, *J* = 8.5 Hz, *Ar*COO), 7.61 (d, 2H, *J* = 8.5 Hz, *Ar*N), 7.26–7.13 (m, 2H, *Ar*F), 7.02–6.97 (m, 2H, *Ar*F), 5.98 (d, 1H, *J* = 4.8 Hz, OH), 4.43–4.40 (m, 2H, CH_2_O), 4.27–4.23 (m, 3H, CH_2_CHOH), 4.11 (t, 2H, *J* = 6.6 Hz, COOCH_2_), 3.46–3.43 (m, 2H, NCH_2_), 3.36–3.30 (m, 1H, CH_2_N), 3.17–3.11 (m, 1H, CH_2_N), 1.65–1.57 (m, 2H, CH_2_CH_2_), 1.43–1.33 (m, 2H, CH_3_CH_2_), 0.91 (t, 3H, *J* = 7.4 Hz, CH_3_); ^13^C NMR (100 MHz, DMSO-*d*_6_), *δ*: 165.2, 153.4, 151.7 (d, *J*_CF_ = 244.0 Hz), 145.6 (d, *J*_CF_ = 10.5 Hz), 144.0, 130.6, 124.9 (d, *J*_CF_ = 3.7 Hz), 122.9, 121.9 (d, *J*_CF_ = 6.8 Hz), 117.3, 116.2 (d, *J*_CF_ = 17.9 Hz), 115.4, 65.9, 64.6, 64.5, 64.2, 49.9, 46.0, 30.5, 18.6, 13.6; MS: for C_23_H_30_FN_2_O_6_ [M+H]^+^ calc. 449.2010 m/z, found 449.3 m/z. 

*3-[2-(4-Fluorophenoxy)ethylamino]-2-hydroxypropyl 4-(Methoxycarbonylamino)benzoate Hydrochloride* (**23a**). Yield 57%; m.p. 193–195°C; HPLC purity 98.99%; UV (nm), *λ*_max⁡_/log⁡⁡*ε* : 271.1/3.49; IR (Zn/Se ATR, cm^−1^): 3323, 2954, 2765, 1745, 1699, 1600, 1230; ^1^H NMR (400 MHz, DMSO-*d*_6_), *δ*: 10.15 (s, 1H, NHCO), 9.35 (bs, 1H, NH), 9.07 (bs, 1H, NH), 7.95 (d, 2H, *J* = 8.5 Hz, *Ar*COO), 7.61 (d, 2H, *J* = 8.5 Hz, *Ar*N), 7.17–7.13 (m, 2H, *m-Ar*F), 7.02–6.98 (m, 2H, *o-Ar*F), 5.97 (s, 1H, OH), 4.30–4.23 (m, 5H, CH_2_CHOH, CH_2_O), 3.69 (s, 3H, CH_3_), 3.40–3.27 (m, 3H, NCH_2_, CH_2_N), 3.13–3.08 (m, 1H, CH_2_N); ^13^C NMR (100 MHz, DMSO-*d*_6_), *δ*: 165.1, 156.8 (d, *J*_CF_ = 236.6 Hz), 154.1 (d, *J*_CF_ = 1.9 Hz), 153.8, 144.0, 130.6, 122.9, 117.3, 116.1 (d, *J*_CF_ = 3.1 Hz), 115.9 (d, *J*_CF_ = 18.3 Hz), 65.9, 64.5, 63.9, 55.9, 49.8, 46.1; MS: for C_20_H_24_FN_2_O_6_ [M+H]^+^ calc. 407.1540 m/z, found 407.3 m/z. 

*3-[2-(4-Fluorophenoxy)ethylamino]-2-hydroxypropyl 4-(Ethoxycarbonylamino)benzoate Hydrochloride* (**23b**). Yield 61%; m.p. 187-188°C; HPLC purity 99.15%; UV (nm), *λ*_max⁡_/log⁡⁡*ε* : 271.1/3.50; IR (Zn/Se ATR, cm^−1^): 3342, 2961, 2748, 1738, 1697, 1597, 1219; ^1^H NMR (400 MHz, DMSO-*d*_6_), *δ*: 10.11 (s, 1H, NHCO), 9.39 (bs, 1H, NH), 9.10 (bs, 1H, NH), 7.94 (d, 2H, *J* = 8.8 Hz, *Ar*COO), 7.61 (d, 2H, *J* = 8.8 Hz, *Ar*N), 7.17–7.12 (m, 2H, *m-Ar*F), 7.02–6.98 (m, 2H, *o-Ar*F), 5.97 (s, 1H, OH), 4.30–4.23 (m, 5H, CH_2_CHOH, CH_2_O), 4.15 (q, 2H, *J* = 7.1 Hz, COOCH_2_), 3.40–3.27 (m, 3H, NCH_2_, CH_2_N), 3.13–3.08 (m, 1H, CH_2_N), 1.25 (t, 3H, *J* = 7.1 Hz, CH_3_); ^13^C NMR (100 MHz, DMSO-*d*_6_), *δ*: 165.2, 156.8 (d, *J*_CF_ = 236.6 Hz), 154.1 (d, *J*_CF_ = 1.9 Hz), 153.3, 144.0, 130.6, 122.9, 117.3, 116.1 (d, *J*_CF_ = 4.0 Hz), 115.9 (d, *J*_CF_ = 19.0 Hz), 65.9, 64.5, 63.9, 60.6, 49.8, 46.1, 14.4; MS: for C_21_H_26_FN_2_O_6_ [M+H]^+^ calc. 421.1697 m/z, found 421.3 m/z. 

*3-[2-(4-Fluorophenoxy)ethylamino]-2-hydroxypropyl 4-(Propoxycarbonylamino)benzoate Hydrochloride* (**23c**). Yield 54%; m.p. 191–193°C; HPLC purity 97.93%; UV (nm), *λ*_max⁡_/log⁡⁡*ε* : 271.1/3.48; IR (Zn/Se ATR, cm^−1^): 3333, 2955, 2762, 1730, 1695, 1598, 1222; ^1^H NMR (400 MHz, DMSO-*d*_6_), *δ*: 10.12 (s, 1H, NHCO), 9.43 (bs, 1H, NH), 9.13 (bs, 1H, NH), 7.94 (d, 2H, *J* = 8.5 Hz, *Ar*COO), 7.62 (d, 2H, *J* = 8.5 Hz, *Ar*N), 7.16–7.12 (m, 2H, *m-Ar*F), 7.01–6.98 (m, 2H, *o-Ar*F), 5.99 (s, 1H, OH), 4.30–4.23 (m, 5H, CH_2_CHOH, CH_2_O), 4.06 (t, 2H, *J* = 6.7 Hz, COOCH_2_), 3.39–3.27 (m, 3H, NCH_2_, CH_2_N), 3.13–3.09 (m, 1H, CH_2_N), 1.69–1.60 (m, 2H, CH_3_CH_2_), 0.93 (t, 3H, *J* = 7.4 Hz, CH_3_); ^13^C NMR (100 MHz, DMSO-*d*_6_), *δ*: 165.2, 156.8 (d, *J*_CF_ = 236.6 Hz), 154.1 (d, *J*_CF_ = 1.9 Hz), 153.5, 144.1, 130.6, 122.9, 117.3, 116.1 (d, *J*_CF_ = 4.5 Hz), 115.9 (d, *J*_CF_ = 19.5 Hz), 66.1, 65.9, 64.5, 63.9, 49.8, 46.1, 21.8, 10.3; MS: for C_22_H_28_FN_2_O_6_ [M+H]^+^ calc. 435.1853 m/z, found 435.3 m/z. 

*3-[2-(4-Fluorophenoxy)ethylamino]-2-hydroxypropyl 4-(Butoxycarbonylamino)benzoate Hydrochloride* (**23d**). Yield 35%; m.p. 180–182°C; HPLC purity 97.48%; UV (nm), *λ*_max⁡_/log⁡⁡*ε* : 271.1/3.50; IR (Zn/Se ATR, cm^−1^): 3334, 2943, 2766, 1731, 1696, 1596, 1226; ^1^H NMR (400 MHz, DMSO-*d*_6_), *δ*: 10.11 (s, 1H, NHCO), 9.40 (bs, 1H, NH), 9.11 (bs, 1H, NH), 7.94 (d, 2H, *J* = 8.6 Hz, *Ar*COO), 7.61 (d, 2H, *J* = 8.6 Hz, *Ar*N), 7.16–7.12 (m, 2H, *m-Ar*F), 7.01–6.98 (m, 2H, *o-Ar*F), 5.98 (s, 1H, OH), 4.30–4.23 (m, 5H, CH_2_CHOH, CH_2_O), 4.10 (t, 2H, *J* = 6.6 Hz, COOCH_2_), 3.40–3.27 (m, 3H, NCH_2_, CH_2_N), 3.14–3.08 (m, 1H, CH_2_N), 1.64–1.57 (m, 2H, CH_2_CH_2_), 1.42–1.33 (m, 2H, CH_3_CH_2_), 0.91 (t, 3H, *J* = 7.4 Hz, CH_3_); ^13^C NMR (100 MHz, DMSO-*d*_6_), *δ*: 165.2,156.8 (d, *J*_CF_ = 236.7 Hz), 154,1 (d, *J*_CF_ = 2.0 Hz), 153.4, 144.0, 130.6, 122.9, 117.3, 116.0 (d, *J*_CF_ = 4.3 Hz), 115.9 (d, *J*_CF_ = 19.3 Hz), 65.9, 64.5, 64.2, 63.9, 49.8, 46.1, 30.5, 18.6, 13.6; MS: for C_23_H_30_FN_2_O_6_ [M+H]^+^ calc. 449.2010 m/z, found 449.3 m/z. 

#### 2.1.2. Lipophilicity Determination by HPLC (Capacity Factor *k*/Calculated log⁡*k*)

A Waters Alliance 2695 XE HPLC separation module and a Waters Photodiode Array Detector 2996 (Waters Corp., Milford, MA, USA) were used. A Symmetry C_18_ 5 *μ*m, 4.6 × 250 mm, part number WAT054275 (Waters Corp., Milford, MA, USA) chromatographic column was used. The HPLC separation process was monitored by Empower 2 Chromatography Data Software, Waters 2009 (Waters Corp., Milford, MA, USA). A mixture of MeOH p.a. (55%) and H_2_O-HPLC–Milli-Q Grade (45%) was used as a mobile phase. The total flow of the column was 1.0 mL/min, injection volume 30 *μ*L, column temperature 45°C, and sample temperature 10°C. The detection wavelength of 210 nm was chosen. The KI methanolic solution was used for the dead time (*t*_*D*_) determination. Retention times (*t*_*R*_) were measured in minutes. The capacity factors *k* were calculated using the Empower 2 Chromatography Data Software according to formula *k* = (*t*_*R*_ − *t*_*D*_)/*t*_*D*_, where *t*_*R*_ is the retention time of the solute, whereas *t*_*D*_ denotes the dead time obtained using an unretained analyte. log⁡⁡*k*, calculated from the capacity factor *k*, is used as the lipophilicity index converted to log⁡⁡*P* scale. The log⁡⁡*k* values of the individual compounds are shown in Table 1.

#### 2.1.3. p*K*_**a**_ Determination

A HPLC system consisting of Merck-Hitachi L-7100 LaChrom (France) pump, UV-Vis detector Shimadzu SPD-10A VP, and integrator Shimadzu C-R8A Chromatopac was used. A Zorbax Eclipse XBD C_18_, 5 *μ*m, 2.1 × 150 mm (Agilent, USA), chromatographic column was used. pH of mobile phase was measured using pH meter Mettler-Toledo MA 235 pH/ion analyzer. To obtain measurable RP-HPLC retention parameters, an addition of organic modifier to the mobile phase is necessary, especially in the case of water-insoluble substances. To determine p*K*_a_, a proper measurement of the eluent pH is a precondition [33]. A mixture of MeOH p.a. (60%) and phosphate buffer (40%) with concentration 0.05 mol/L was used as a mobile phase. The pH of the mobile phase ranged at 6.10–9.70. pH was measured after mixing the aqueous buffer and MeOH. The electrode system was calibrated with the usual aqueous buffers [34]. The flow rate of the column was 0.25 mL/min; the injection volume was 5 *μ*L (methanolic solution with concentration 0.5 mmol/L); the column temperature was 25°C. The detection wavelength of 254 nm was chosen. MeOH was used for the dead time (*t*_*D*_) determination. Retention times (*t*_*R*_) were measured in minutes. Measurement of each compound in each of mobile phases was repeated four times. The capacity factors *k* were calculated using the formula *k* = (*t*_*R*_ − *t*_*D*_)/*t*_*D*_, where *t*_*R*_ is the retention time of the solute, whereas *t*_*D*_ denotes the dead time obtained using an unretained analyte [35]. p*K*_a_ was measured using the dependence of the retention factor on the pH of the mobile phase. This relationship was fitted by a sigmoid curve, where p*K*_a_ is the value of pH in the sigmoid inflection point [34]. Values of p*K*_a_ obtained for water MeOH/buffer mixture were recalculated for water medium [33, 36, 37]. The p*K*_a_ values of individual compounds are shown in Table 1.

#### 2.1.4. Calculations of Lipophilicity and Other Molecular Descriptors

All values of molecular descriptors were calculated for the uncharged molecules. log⁡⁡*P* values (*i.e.,* the logarithm of the partition coefficient for octanol/water) were predicted using ACD/Percepta software (ACD/Labs, ver. 12.01, Advanced Chemistry Development, Inc., Toronto, ON, Canada, 2012). log⁡⁡*S* values (as aqueous log⁡⁡*S* at pH 7.4) were calculated by ACD/Percepta software (ACD/Labs, ver. 12.01, Advanced Chemistry Development, Inc., Toronto, ON, Canada, 2012). ACD/Percepta calculates aqueous solubility values at any pH under the standard conditions (and zero ionic strength). The accuracy of calculations (according to the vendor) for simple structures is usually better than 0.2–0.5 logarithmic units (for complex structures it is better than 0.5–1.0 logarithmic units). Solubility is not derived from log⁡⁡*P* and takes into account not only the pH (solubility as a function of pH) but also compares the fragmental estimations with the experimental material from ca 6000 compounds databased. Molar volume (MV [cm^3^]) and surface tension (ST [dyne/cm]) were calculated by means of ACD/Percepta (ACD/Labs, ver. 12.01, Advanced Chemistry Development, Inc., Toronto, ON, Canada, 2012). All the results are shown in Table 1.

### 2.2. Biology

#### 2.2.1. *In Vitro* Antimycobacterial Evaluation

Clinical isolates of *Mycobacterium avium *subsp.* paratuberculosis* CIT03 and *M. intracellulare *were grown in Middlebrook broth (MB), supplemented with oleic-albumin-dextrose-catalase supplement (OADC, Becton Dickinson, UK) and mycobactin *J* (2 *μ*g/mL). Identification of these isolates was performed using biochemical and molecular protocols. At log phase growth, the culture (10 mL) was centrifuged at 15,000 rpm/20 min using a bench top centrifuge (Model CR 4–12, Jouan Inc., UK). Following the removal of the supernatant, the pellet was washed in fresh Middlebrook 7H9GC broth and resuspended in fresh supplemented MB (10 mL). The turbidity was adjusted to match McFarland standard no. 1 (3 × 10^8^ cfu) with MB broth. A further 1 : 20 dilution of the culture was then performed in MB broth. The antimicrobial susceptibility of all four mycobacterial species was investigated in a 96-well plate format. In these experiments, sterile deionised water (300 *μ*L) was added to all outer-perimeter wells of the plates to minimize evaporation of the medium in the test wells during incubation. Each evaluated compound (100 *μ*L) was incubated with each of the mycobacterial species (100 *μ*L). Dilutions of each compound were prepared in duplicate. For all synthesized compounds, final concentrations ranged from 1,000 *μ*g/mL to 8 *μ*g/mL. All compounds were prepared in DMSO, and subsequent dilutions were made in supplemented MB. The plates were sealed with parafilm and incubated at 37°C, for 7 days in the case of *M. intracellulare *and 11 days in the case of *M. avium paratuberculosis*. Following incubation, a 10% addition of alamarBlue (AbD Serotec) was mixed with each well, and readings at 570 nm and 600 nm were taken, initially for background subtraction and subsequently after 24 h reincubation. The background subtraction is necessary for strongly coloured compounds, where the colour may interfere with the interpretation of any colour change. For noninterfering compounds, a blue colour in the well was interpreted as an absence of growth, and a pink colour was scored as growth. The MIC was initially defined as the lowest concentration which prevented a visual colour change from blue to pink. The standards ciprofloxacin (CPX), isoniazid (INH), pyrazinamide (PZA), and rifampicin (RIF) are clinically used as antimycobacterial drugs. The MIC for mycobacteria was defined as a 90% or greater (IC_90_) reduction of growth in comparison with the control. The MIC/IC_90_ value is routinely and widely used in bacterial assays and is a standard detection limit according to the Clinical and Laboratory Standards Institute (CLSI, http://www.clsi.org/).

#### 2.2.2. Study of Photosynthetic Electron Transport (PET) in Spinach Chloroplast

Chloroplasts were prepared from spinach (*Spinacia oleracea* L.) according to Masarovičová and Král'ová [38]. The inhibition of photosynthetic electron transport (PET) in spinach chloroplasts was determined spectrophotometrically (Genesys 6, Thermo Scientific, USA), using an artificial electron acceptor 2,6-dichlorophenol-indophenol (DCIPP) according to Král'ová et al. [39], and the rate of photosynthetic electron transport was monitored as a photoreduction of DCPIP. The measurements were carried out in phosphate buffer (0.02 mol/L, pH 7.2) containing sucrose (0.4 mol/L), MgCl_2_ (0.005 mol/L), and NaCl (0.015 mol/L). The chlorophyll content was 30 mg/L in these experiments, and the samples were irradiated (~100 W/m^2^ with 10 cm distance) with a halogen lamp (250 W) using a 4 cm water filter to prevent warming of the samples (suspension temperature 22°C). The studied compounds were dissolved in DMSO due to their limited water solubility, and the concentration of DMSO was constant (8% (v/v)) in control as well as in amphiphile-treated samples.

#### 2.2.3. *In Vitro* Cytotoxicity Assay

The human monocytic leukaemia THP-1 cell line was purchased from the European Collection of Cell Cultures (ECACC, Salisbury, UK). Cells were cultured in RPMI 1640 medium supplemented with 10% fetal bovine serum, 2% L-glutamine, 1% penicillin, and streptomycin at 37°C with 5% CO_2_. Cells were passaged at approximately one-week intervals. Cells were routinely tested for the absence of mycoplasma (Hoechst 33258 staining method). RPMI 1640 culture medium, PBS, penicillin, and streptomycin were purchased from Lonza (Verviers, Belgium); FBS was acquired from Sigma-Aldrich. THP-1 cells were exposed to compounds dissolved in DMSO in concentrations ranging from 1.1 to 30 *μ*mol/L at 37°C in RPMI 1640 medium for 24 h. The maximum concentration of DMSO in the assays never exceeded 0.1%. Cytotoxicity of the compounds was determined using a WST-1 assay kit (Roche Diagnostics, Mannheim, Germany), as described previously [40, 41]. The median lethal dose values, LD_50_, were deduced through the production of a dose-response curve. All data from three independent experiments were evaluated using GraphPad Prism 5.00 software (GraphPad Software, San Diego, CA, USA, http://www.graphpad.com).

## 3. Results and Discussion

### 3.1. Chemistry

#### 3.1.1. Synthesis

**Scheme 1:**
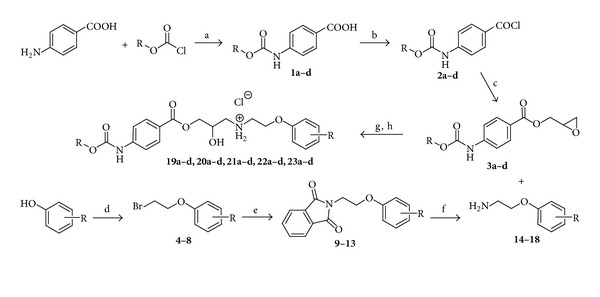
Synthesis of evaluated compounds. Reagents and conditions: (a) acetone, pyridine; (b) SOCl_2_, toluene; (c) 2,3-epoxypropan-1-ol, THF, and TEA; (d) 1,2-dibromoethane, NaOH; (e) potassium phthalimide, KI, and DMF; (f) NH_2_NH_2_·H_2_O, ethanol; (g) propan-2-ol; (h) HCl, Et_2_O.

All the studied compounds were prepared by multiple-step reaction described in Scheme 1. Epoxides **3a**–**d** were prepared from 4-aminobenzoic acid using reaction with methyl-, ethyl-, propyl-, and butyl-chloroformates giving appropriate acids **1a**–**d**. Chlorides **2a**–**d** formed by thionyl chloride treatment gave desired epoxides after reaction with 2,3-epoxypropan-1-ol. The oxirane ring was opened by addition of methoxy- or fluorosubstituted phenoxyethylamines **14**–**18** prepared by Gabriel synthesis via intermediates **4**–**8** and **9**–**13**. Acquired bases were transformed to hydrochloride salts with higher water solubility.

#### 3.1.2. Physicochemical Properties

**Figure 1:**
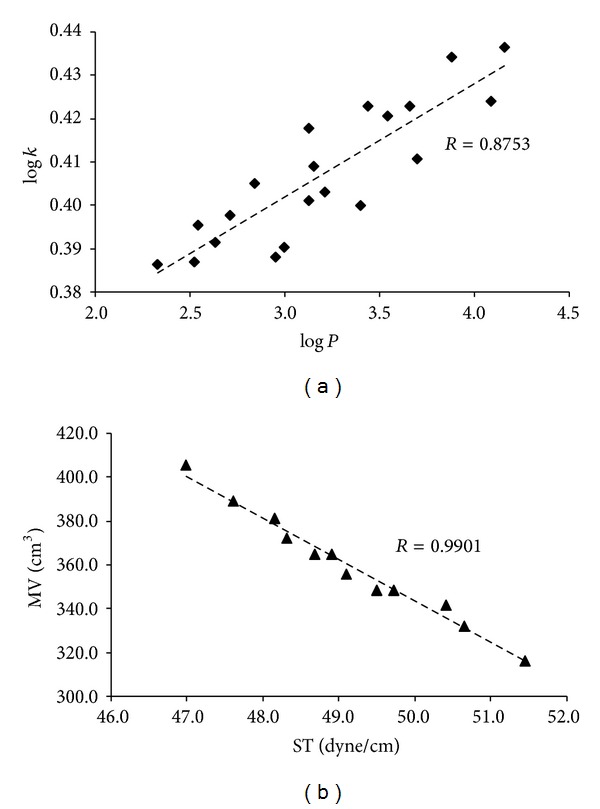
Comparison/match of experimentally found log⁡⁡*k* values with calculated log⁡⁡*P* of uncharged molecules (a); match of calculated data for uncharged molecules of molar volume with surface tension (b).

Lipophilicity of the studied compounds was determined by RP-HPLC as capacity factor logarithm (log⁡⁡*k*) and calculated as log⁡⁡*P* for the uncharged molecules using ACD/Percepta software. The results for 2-hydroxy-3-[(2-aryloxyethyl)amino]propyl 4-[(alkoxycarbonyl) amino]benzoates **19a**–**23d** are shown in Table 1 and illustrated in Figure 1(a).

The results obtained with all the compounds show that the experimentally determined lipophilicities (log⁡⁡*k*) of the discussed compounds are relatively in accordance with the calculated log⁡⁡*P* values of uncharged compounds **19a**–**23d** as shown in Figure 1(a). The influence of R^1^ substituents on lipophilicity is as follows: CH_3_ < C_2_H_5_ < C_3_H_7_ < C_4_H_9_. Within the individual series lipophilicity determined as log⁡⁡*k* values increased as follows: 2,6-OCH_3_ < 2-OCH_3_ < 4-OCH_3_ < 2-F < 4-F. Generally, it could be concluded that the prediction power of the used experimental log⁡⁡*k* or calculated log⁡⁡*P* (ACD/Percepta) values for extrapolation of transport modifications may be a good tool for searching potential drugs, namely, in the range of interpolation.

In the current investigation, the experimentally determined dissociation constants (p*K*_a_ data) as well as solubility/polarity (log⁡⁡*S*), molar volume (MV), and surface tension (ST) of all compounds calculated for the uncharged molecules by ACD/Percepta were examined to determine if these factors play a role in their biological activities. All the target compounds are strong bases according to determined p*K*_a_ values, whereas 2,6-OCH_3_ and 2-OCH_3_ substituted compounds show the highest basicity, while fluorosubstituted compounds showed the lowest basicity. According to solubility/polarity (log⁡⁡*S*), it can be stated that all the compounds are moderately or poorly aqueous soluble, whereas dependence on substituent R^2^ is similar as described above. 2,6-Dimethoxy or 2-methoxy substituted compounds (derivatives with the lowest lipophilicity) show the best water solubility, while fluorosubstituted compounds (compounds with the highest lipophilicity) possess the lowest solubility. Within individual series, solubility decreases from methyl to butyl chain. A linear match can be observed for the dependence between molar volume and surface tension (Figure 1(b)). It can be stated that compound 2-hydroxy-3-[2-(2,6-dimethoxyphenoxy)ethylamino]-propyl 4-(butoxycarbonylamino)benzoate hydrochloride (**21d**) which expresses the lowest surface tension also possesses the highest molar volume and medium value of basicity and lipophilicity (Table 1).

### 3.2. Biology

#### 3.2.1. *In Vitro* Antimycobacterial Evaluation

All the compounds were evaluated for their *in vitro* antimycobacterial activity against atypical mycobacterial strains *Mycobacterium avium *subsp.* paratuberculosis* CIT03 and *M. intracellulare*. These pathogens can occur in immunocompromised patients in whom they can cause various pulmonary or gastrointestinal diseases. Both strains were chosen due to their resistance to standard antimycobacterial therapy; therefore, isoniazid, pyrazinamide and rifampicin (as first-line antituberculotic drugs), and ciprofloxacin (as an alternative antituberculotic/antimycobacterial drug) [42, 43] were used as standards. As shown in Table 1, isoniazid and pyrazinamide were inactive. The target compounds showed a wide range of activities as shown in Table 1. Nevertheless, the 12 compounds expressed higher antimycobacterial activities than standard ciprofloxacin. Most of the compounds were active against *M. avium paratuberculosis*. 2-Hydroxy-3-[2-(2,6-dimethoxyphenoxy)ethylamino]propyl 4-(butoxycarbonylamino)benzoate hydrochloride (**21d**), 2-hydroxy-3-[2-(2-methoxyphenoxy)ethylamino]propyl 4*-*(butoxycarbonylamino)benzoate hydrochloride (**19c**), and 2-hydroxy-3-[2-(4-methoxyphenoxy)ethylamino]propyl 4-(butoxycarbonylamino)benzoate hydrochloride (**20d**) showed the highest activity against *M. avium *subsp.* paratuberculosis*, and **21d**, **20d,** and 3-[2-(2-fluorophenoxy)ethylamino]-2-hydroxypropyl 4-(ethoxy-carbonylamino)benzoate hydrochloride (**22b**) expressed the highest activity against *M. intracellulare*.

It can be generally concluded that activities against both strains increase with lipophilicity (see Table 1), that is, butyl **d** > propyl **c** > ethyl **b** > methyl **a**. This observation can be attributed to the decrease in activity with increasing polarity within individual series. Also, the increase of antimycobacterial effect is related to the increase in the bulkiness of individual R^1^ substituents and with the decrease of calculated surface tension (*i.e.,* with the increase of surface activity) within the series of compounds. Although the discussed compounds did not express significant antimycobacterial activity, based on the results it can be stated that the most active compounds possess the lowest calculated surface tension and the highest molar volume. Simultaneously, they have the highest lipophilicity and the lowest polarity within individual series. Therefore, it can be concluded that R^1^ substitution (alkyl chain) has a dominant influence on the activity, while R^2^ substituent plays only a secondary role. Nevertheless, for the activity minimal critical alkyl chain length is necessary (butyl and in some cases propyl). One of the possible mechanisms of action of this type of compounds may be based on perturbation of the biological membrane and an effect on the enzymatic system within *Mycobacterium*, that is, inhibition of biosynthesis of various mycobacterial components [44].

#### 3.2.2. Acceleration of Photosynthetic Electron Transport (PET) in Spinach Chloroplasts

**Figure 2:**
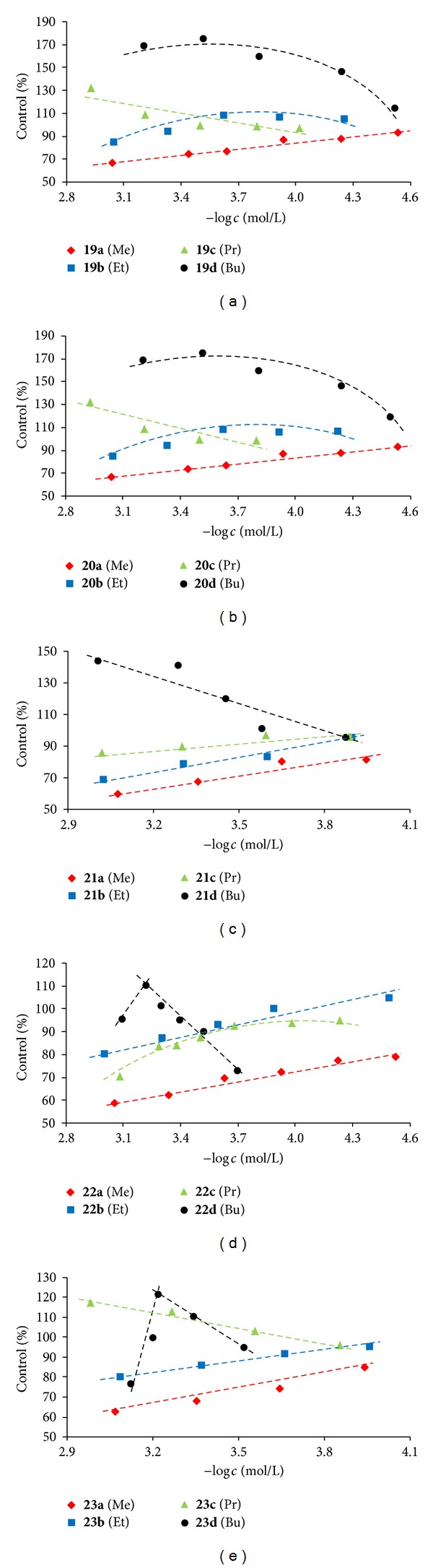
Dependence of the rate of photosynthetic electron transport (expressed in % of the control) on the negative logarithm of compound concentration: **19a**–**d** (a); **20a**–**d** (b); **21a**–**d** (c); **22a**–**d** (d); **23a–d** (e).

Due to limited aqueous solubility of the amphiphilic compounds tested, their effect on PET was studied in the suspension of spinach chloroplasts containing a constant DMSO concentration (8% v/v) both in the control as well as in amphiphile-treated samples. All the results are summarized in Figure 2. From the dependences of the rate of PET on compound concentration (log⁡⁡*c*), see Figure 2, it can be concluded that all butyl derivatives (**19d**, **20d**, **21d**, **22d**, and **23d**) significantly stimulated the rate of PET (*i.e.,* oxygen evolution rate). In addition, relatively strong PET stimulation was observed with some propyl derivatives (**19c**, **20c**), while PET accelerating effects of methyl and ethyl derivatives were generally low. Acceleration of PET indicates that the above-mentioned compounds can function as uncouplers of photosynthesis, uncoupling ATP synthesis from photosynthetic electron flow. In terms of the chemiosmotic hypothesis, an uncoupler increases the permeability of membranes to protons, thereby dissipating the electrochemical proton gradient [45]. The stimulating effect of membrane active compounds on the rate of PET may be caused by an increase in the permeability of chloroplast envelope membrane or its destruction, resulting in the restraint of the phosphorylation system [26, 27].

Based on the structure of tested compounds (relative large size of the molecule and high polarity of the spacer between two benzene rings), it is not probable that these compounds can cross the thylakoid membrane and act as protonophores or form a channel through the lipid membrane which would be suitable for proton transport. On the other hand, their amphiphilic structure as well as the fact that the highest PET accelerating effects were obtained with butyl derivatives suggest that these compounds can affect thylakoid membrane integrity leading to uncoupling of phosphorylation from electron transport. These compounds probably induce conformational changes in biomembranes, increasing their permeability and enabling ion leakage. Similar PET stimulating effects in chloroplasts were observed with Triton and other detergents [46], sodium dodecyl sulphate [27] and surfactants of the quaternary ammonium salt type [47, 48]. In the paper by Šeršeň and Lacko (1995), for characterization of the arrangement of untreated as well as surfactant-treated thylakoid membranes, the order parameter S was calculated from EPR spectrum of the spin label CAT 16 (*N*-hexadecyl-*N*-tempoyl-*N,N*-dimethylammonium bromide) incorporated into thylakoid membranes. At certain concentrations of 1-alkyl-1-ethylpiperidinium bromides, an enhancement of both S and OER above their values in control samples was observed. This indicated that the stimulating effect of these cationic surfactants on OER in chloroplasts could be caused by changes in arrangement of thylakoid membranes.

#### 3.2.3. *In Vitro* Cytotoxicity Assay

**Figure 3:**
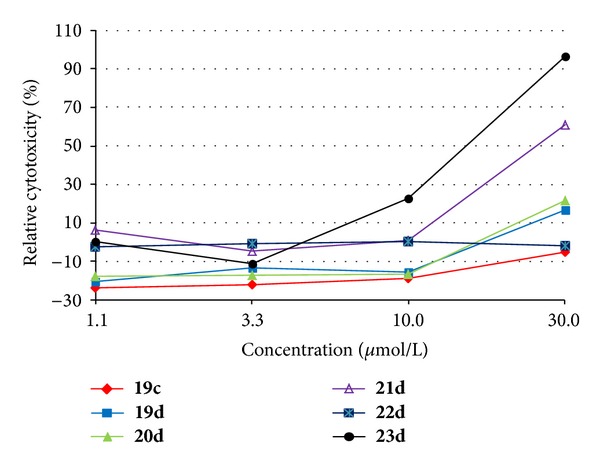
Cytotoxicity of tested compounds **19c**, **19d**, **20d**, **21d**, **22d**, and **23d** against human THP-1 cells after 24 h incubation.

The most effective compounds in relation to antimycobacterial activity **19c**, **19d**, **20d**, **21d**, **22d**, and **23d** were tested for their *in vitro* cytotoxicity LD_50_ (*μ*mol/L) using human monocytic leukaemia THP-1 cells [40]. All the results are illustrated in Figure 3. In several previous studies, the toxicity of tested compounds (including antibacterial agents, e.g., [41, 49]) was also assessed on THP-1 cells. LD_50_ (lethal dose 50%) represents the dose required to kill half the members of a tested population. The highest dose of all tested compounds in the medium was 30 *μ*mol/L, which was the limit of solubility for many compounds in that insoluble aggregates were observed. However, in spite of insolubility, an increase in LD_50_ relative to cytotoxicity was detected for 2,6-OCH_3_ substituted **21d** (61%) and 4-F substituted **23d** (96%) compounds. The LD_50_ values against the human monocytic leukaemia THP-1 cell line were determined to be >10 *μ*mol/L for all tested compounds. Based on these observations, it can be concluded that the discussed compounds did not express any increased toxicity (e.g., LD_50_ of oxaliplatin and camptothecin were much lower: 1.7 ± 0.6 *μ*mol/L and 0.16 ± 0.07 *μ*mol/L, resp.), and thus, compound **20d** can be considered as a promising agent for subsequent design of novel antimycobacterial agents.

## 4. Conclusion

A series of twenty substituted 2-hydroxy-3-[(2-aryloxyethyl)amino]propyl 4-[(alkoxycarbonyl)amino]benzoates were prepared and characterized. The prepared compounds were tested for their ability to stimulate photosynthetic electron transport (PET) in spinach chloroplasts (*Spinacia oleracea* L.) by uncoupling ATP synthesis from photosynthetic electron flow and for their antimycobacterial activity. It can be concluded that all butyl derivatives (**19d**, **20d**, **21d**, **22d**, and **23d**) significantly stimulated the rate of PET, and a relatively strong PET stimulation was also observed with some propyl derivatives (**19c**, **20c**), while PET accelerating effects of methyl and ethyl derivatives were generally low. PET stimulation by studied amphiphiles could be connected with conformational changes in thylakoid membranes resulting in an increase of their permeability and subsequent uncoupling of phosphorylation from electron transport. Within the series of the compounds, 2-hydroxy-3-[2-(2,6-dimethoxyphenoxy)ethylamino]propyl 4-(butoxycarbonylamino)benzoate hydrochloride (**21d**), 2-hydroxy-3-[2-(2-methoxyphenoxy)ethylamino]-propyl [4-(butoxycarbonylamino)benzoate] hydrochloride (**19c**), and 2-hydroxy-3-[2-(4-methoxyphenoxy)ethylamino]propyl 4-(butoxycarbonylamino)benzoate hydrochloride (**20d**) showed the highest activity against *M. avium paratuberculosis*, and **21d**, **20d**, and 3-[2-(2-fluorophenoxy)ethylamino]-2-hydroxypropyl 4-(ethoxy-carbonylamino)benzoate hydrochloride (**22b**) expressed the highest activity against *M. intracellulare*. The most active compounds possess the lowest calculated surface tension (*i.e.,* the highest surface activity) and the highest molar volume, and simultaneously they have the highest lipophilicity and the lowest polarity within individual series. The LD_50_ values against the human monocytic leukaemia THP-1 cell line were determined to be >10 *μ*mol/L for all tested compounds; thus, the compound **20d** can be considered as a promising agent for subsequent design.

## Figures and Tables

**Table 1:** Structure of the target compounds **19a**–**23d** and comparison of calculated lipophilicities (log *P*) with determined log *k* values; calculated values of solubility (log *S*), molar volume (MV [cm^3^]), surface tension (ST [dyne/cm]), and determined p*K*_a_ values, and *in vitro* antimycobacterial activity (MIC [*μ*mol/L]) of compounds compared to ciprofloxacin (CPX), isoniazid (INH), pyrazinamide (PZA), and rifampicin (RIF) standards.

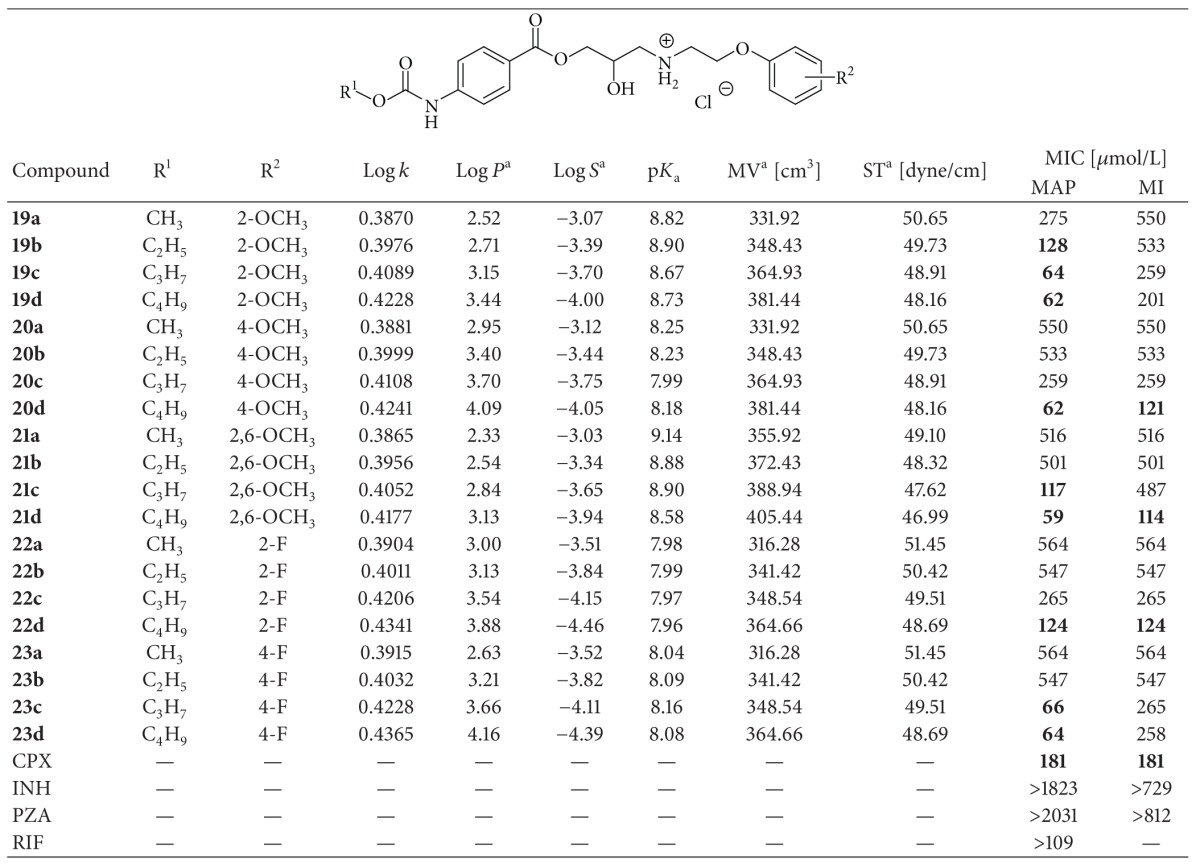

^a^Calculated for the uncharged molecule; MAP: *Mycobacterium avium* subsp. *paratuberculosis* CIT03; MI: *Mycobacterium intracellulare*.
